# Cleaning and disinfection of surfaces in hospitals. Improvement in quality of structure, process and outcome in the hospitals in Frankfurt/Main, Germany, in 2016 compared to 2014

**DOI:** 10.3205/dgkh000312

**Published:** 2018-07-17

**Authors:** Angelika Hausemann, Miriam Grünewald, Ulla Otto, Ursel Heudorf

**Affiliations:** 1 Public Health Department of the City of Frankfurt/Main, Germany

**Keywords:** hospital hygiene, surface cleaning, surface disinfection, infection control visits, public health department

## Abstract

The cleaning and disinfection of surfaces in hospitals is becoming increasingly important in the multi-barrier approach for preventing infection, in addition to hand hygiene and proper reprocessing of medical devices. Therefore, in 2014, the quality of structure, process and outcome of surface preparation was checked in all hospitals in Frankfurt/Main, Germany. Because of great need for improvements, this monitoring was repeated in 2016. The data are presented in comparison to those in 2014.

**Methods:** All 16 hospitals provided information on the quality of structure. Data on quality of process was obtained through direct observation during cleaning and disinfection of rooms and their bathrooms. Data on quality of result was acquired using the fluorescence method, i.e., marking surfaces with a fluorescent liquid and testing whether this mark has been sufficiently removed by cleaning. The results are compared to those of the 17 hospitals monitored in 2014, before the closing of one of the hospitals**.**

**Results:** Quality of structure [data from 2014]: In all hospitals, the employees were trained regularly. In 14 (88%) [12; 71%] of those, the foremen had the required qualifications. In 1 (6%) [6; 35%] hospitals, some uncertainty remained concerning the interface of the cleaning and nursing care services. A complete cleaning was reported to take place in 12 (75%) [12; 70%] hospitals on Saturdays and in 4 (25%) [2; 11%] hospitals on Sundays. Quality of process: During process monitoring, the different surfaces with frequent hand or skin contact were prepared to different extents (91–100%) [70–100%]. Quality of result: 88% [75%] of fluorescent marks were appropriately removed.

**Conclusion:** Compared to 2014, a clear improvement were seen in 2016, especially in the qualification of the foremen and in terms of clearly defining the interface between cleaning and care services as well as the quality of process and outcome. Nevertheless, regarding the growing importance of proper reprocessing of hospital surfaces for prevention of infections and/or colonizations, further improvements are mandatory, including a program for better education of the cleaning staff.

## Zusammenfassung

Neben der Händehygiene und der Instrumentenaufbereitung kommt der Flächenreinigung und -desinfektion in Kliniken eine wichtige Bedeutung in der Verhinderung von Erregerübertragungen und damit auch Infektionen zu. Nachdem bei einer umfassenden, standardisierten Erhebung der Struktur-, Prozess- und Ergebnisqualität der Flächenaufbereitung der Frankfurter Kliniken im Jahr 2014 deutlicher Verbesserungsbedarf gefunden wurde, wurde diese Erhebung im Jahr 2016 mit identischer Methode wiederholt.

**Methoden:** Wie in 2014 wurden auch in 2016 alle Krankenhäuser im Hinblick auf die Strukturqualität (incl. Verträge mit Reinigungsfirmen, Qualitätsanforderungen, vereinbarte Zeitkontingente etc.) und die Prozess- und Ergebnisqualität überwacht. Da inzwischen ein Haus geschlossen hat, betraf dies jetzt insgesamt 16 Häuser (2014: 17). Vor Ort wurde die Reinigung in jeweils mindestens 5 Zweibettzimmern pro Krankenhaus beobachtet und die Ergebnisqualität an jeweils 15 vorab standardisiert angebrachten fluoreszierenden Markierungen (Glow-Check) erhoben.

**Ergebnisse:** Strukturqualität [Werte aus 2014]: In 14 (88%) Kliniken hatten die Vorarbeiter die geforderte Qualifikation [2014: 12; 71%]. In 15 (94%) Kliniken gab es Schnittstellenpläne, d.h. genaue Angaben für den Reinigungs- und Pflegedienst [11; 65%]. Eine „volle Reinigungsleistung“ an Samstagen wurde in 12 (75%) Kliniken [12; 71%], an Sonntagen in 4 (25%) Kliniken angegeben [2; 12%]. Die vertraglich vorgegebene Reinigungsleistung (beobachtete Leistung in Klammern) lag im Mittel bei 168 m^2^/h (155 m^2^/h) pro Patientenzimmer [178 und 148 m^2^/h] und bei 63 m^2^/h (34 m^2^/h) pro Nasszelle [69 und 33 m^2^/h]. Bei der Beobachtung der Prozessqualität wurden die verschiedenen Handkontaktflächen im Jahr 2016 besser aufbereitet. Bei der Prüfung der Ergebnisqualität: wurden 88% der erreichbaren Punkte erreicht [2014: 75%].

**Schlussfolgerung:** Im Vergleich zu 2014 zeigte sich 2016 eine deutliche Verbesserung, insbesondere in den Häusern bzw. in den Bereichen, bei welchen zuvor ein höherer Verbesserungsbedarf festgestellt worden war. Die Häuser haben teilweise erhebliche Anstrengungen unternommen – u.a. bei der Schulung des Personals und der Kontrolle der Reinigung. Gleichwohl werden immer wieder teilweise auch erhebliche Fehler beobachtet und es erreichen uns Patientenbeschwerden wegen Reinigungsmängeln, d.h. weitere Verbesserungen sind erforderlich.

## Introduction

Good hygiene is crucial for the prevention of infections and pathogen transmission in the hospital. With more older, multimorbid patients and shorter hospital stays, more invasive methods in diagnostics and therapy, and more medical devices, the risk of infections has increased during the past years. 

Cleaning and disinfection of surfaces in the hospital is becoming increasingly important in the multi-barrier approach for preventing infection, in addition to hand hygiene and proper reprocessing of medical devices. With new publications available, previous controversial discussions [1] as to the role of surface disinfection have declined. The relevance of surface disinfection is being increasingly accepted. In their review, Gebel at al. [2] stated:

- “There is good evidence that contaminated dry surfaces contribute to the spread of nosocomial pathogens.
- It is undisputed that environmental disinfection is necessary in certain risk areas and in outbreak situations.
- It is widely acknowledged that proper use of disinfectants contributes to the control of pathogens in outbreak situation as part of a bundle strategy.”

In the meantime, several national guidelines and recommendations have become available [3], [4].

In Germany, the national Hospital Hygiene and Infection Prevention Commission KRINKO emphasizes the multi-barrier approach, defines surfaces and areas with different transfer risks, and attaches particular importance to the regular and appropriate disinfection of the surfaces with frequent hand and skin contact [3]. The commission emphasizes the importance of proper training and instruction, as well as the supervision of staff, and the need to “provide sufficient time for the work to be carried out properly”. However, the commission does not itself provide any defined training requirements or reference values for the surfaces to be cleaned per unit time.

Further support is provided by the German Society for Hospital Hygiene (DGKH) in its “Hygienic Criteria for the Cleaning Service” from 2013 [5], especially with regard to the selection and commissioning of cleaning service providers. Requirements for the certification of the service providers and basic training of the managers according to the guidelines of GUV-R 107-002 and GUV-R 101-10 have been published [6], [7]. 

Regarding time allotted for cleaning and disinfection of defined surfaces (m^2^/h), current guidelines have been published by the RAL-Gütegemeinschaft Gebäudereinigung e.V. in 2011 [8]: In the patient rooms in hospitals, 130–220 m^2^ should be reprocessed per hour, and in toilets, showers and baths 60–120 m^2^/h. 

Based on these guidelines, the public health department in Frankfurt/Main, Germany, has monitored cleaning and disinfection of surfaces in the hospitals of Frankfurt for many years. In 2014, the systematic monitoring of quality of structure, process, and outcome of surface preparation of all hospitals in Frankfurt/Main exhibited a great need for improvement [9]. Therefore, in 2016, this monitoring was repeated. The data are presented here in comparison to those in 2014.

## Materials and methods

Based on the recommendations of the KRINKO and of the DGKH [3], [5] the structural, process, and outcome quality of cleaning and disinfection in all Frankfurt hospitals was monitored and compared to the data of 2014. In the interim, one hospital has closed, so that in 2016, 16 hospitals and in 2014, 17 hospitals were monitored.

### Structural quality

As part of a questionnaire-based survey, data was obtained regarding the qualification of the foreman, staff training and quality control, the interface of the responsibilities of house cleaning and nursing personnel, the work instructions (standard operating procedures SOP), the cleaning performance on weekends, and time commissioned for the preparation of a two-bed room as well as a bathroom. The reprocessing of the beds was not the subject of the survey.

### Process quality

On the day of the control visit, reprocessing of at least 5 two-bed rooms and bathrooms was monitored in every hospital. Reprocessing was surveyed by employees of the Public Health Department and recorded in detail, including the time consumed.

### Outcome quality 

Before the visit, hygiene personnel of the respective hospitals had marked definite points in fluorescent ink, according to the CDC’s recommendation [10]. During the control visit, if and how these points had been removed by cleaning was determined using an ultraviolet flashlight. Complete removal of the mark was scored as two points, partial removal was given one point, and zero points were awarded if the mark was still visible, i.e. this area had not been processed.

## Results

### Structural quality

Table 1 (Tab. 1) shows the data of structural quality. Compared to 2014, more hospitals provided a plan for cleaning and disinfection with detailed definitions of the tasks of the cleaning service (usually all surfaces) and nursing care service (usually surfaces of medical devices). Two more hospitals had trained their foreman properly and had increased the range of cleaning on Sundays, now covering complete cleaning (comparable to normal working days). Most of the hospitals had implemented internal routine control via fluorescent markers.

In Table 2 (Tab. 2), the cleaning performance, expressed as m^2^ per hour, recommended by the RAL is depicted and compared to data of the DGKH in 2013 (questionnaire survey) and the on-site surveys in Frankfurt hospitals 2014 and 2016. The commissioned m^2^ per hour for the cleaning of the patient rooms and bathrooms in Frankfurt/Main were within the range indicated in the RAL recommendation. However, the performance for the cleaning of the bathrooms was very much lower than commissioned – with no significant differences between 2014 and 2016.

### Process quality

**Figure 1:**
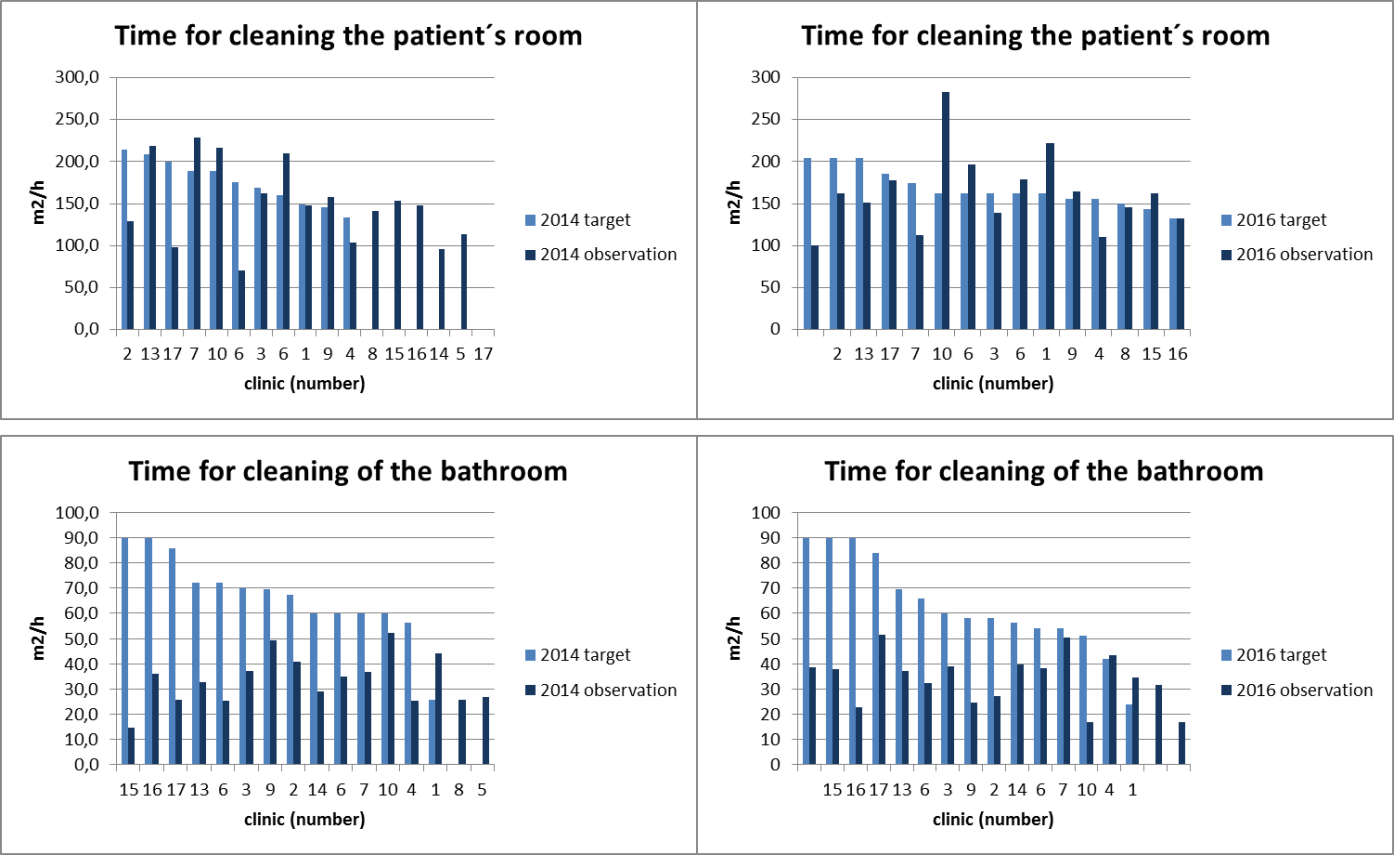
Time commissioned and time needed for the reprocessing of patients’ rooms and bathrooms in the hospitals in Frankfurt/Main – 2016 vs 2014

Time commissioned and time required for the reprocessing of surfaces varied widely between the hospitals (Figure 1 (Fig. 1)) – in 2016 as well as in 2014. In the patients’ rooms in most hospitals, the observations corresponded to the commissioned time, but in the bathrooms, performance was much lower than commissioned – in all hospitals and in 2016 as well as in 2014.

**Figure 2:**
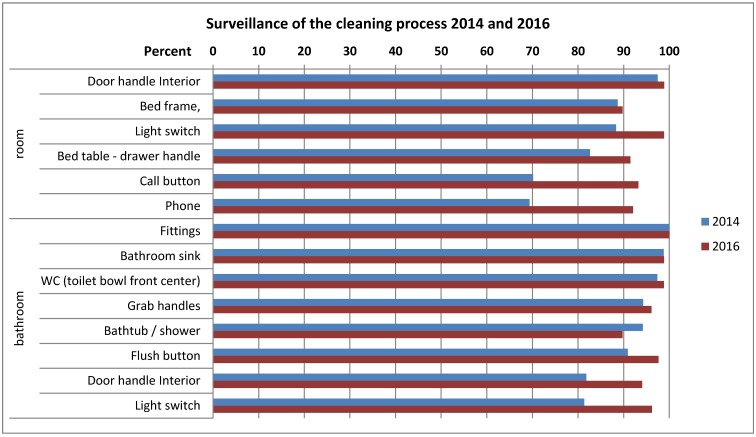
Monitoring quality of process of the cleaning and disinfection in hospitals in Frankfurt/Main, 2014 and 2016, by visual observation according to the different sites – percent of properly cleaned sites

Figure 2 (Fig. 2) shows that in 2016 the processing of door handles, bedframe light switch, etc. was done properly in more than 90% of the rooms. Compared to 2014, improvements could be seen especially at those sites which had previously been reprocessed insufficiently, i.e., the call button, phone, drawer handle of the bed table, and door handle and light switch in the bathroom.

### Outcome quality

**Figure 3:**
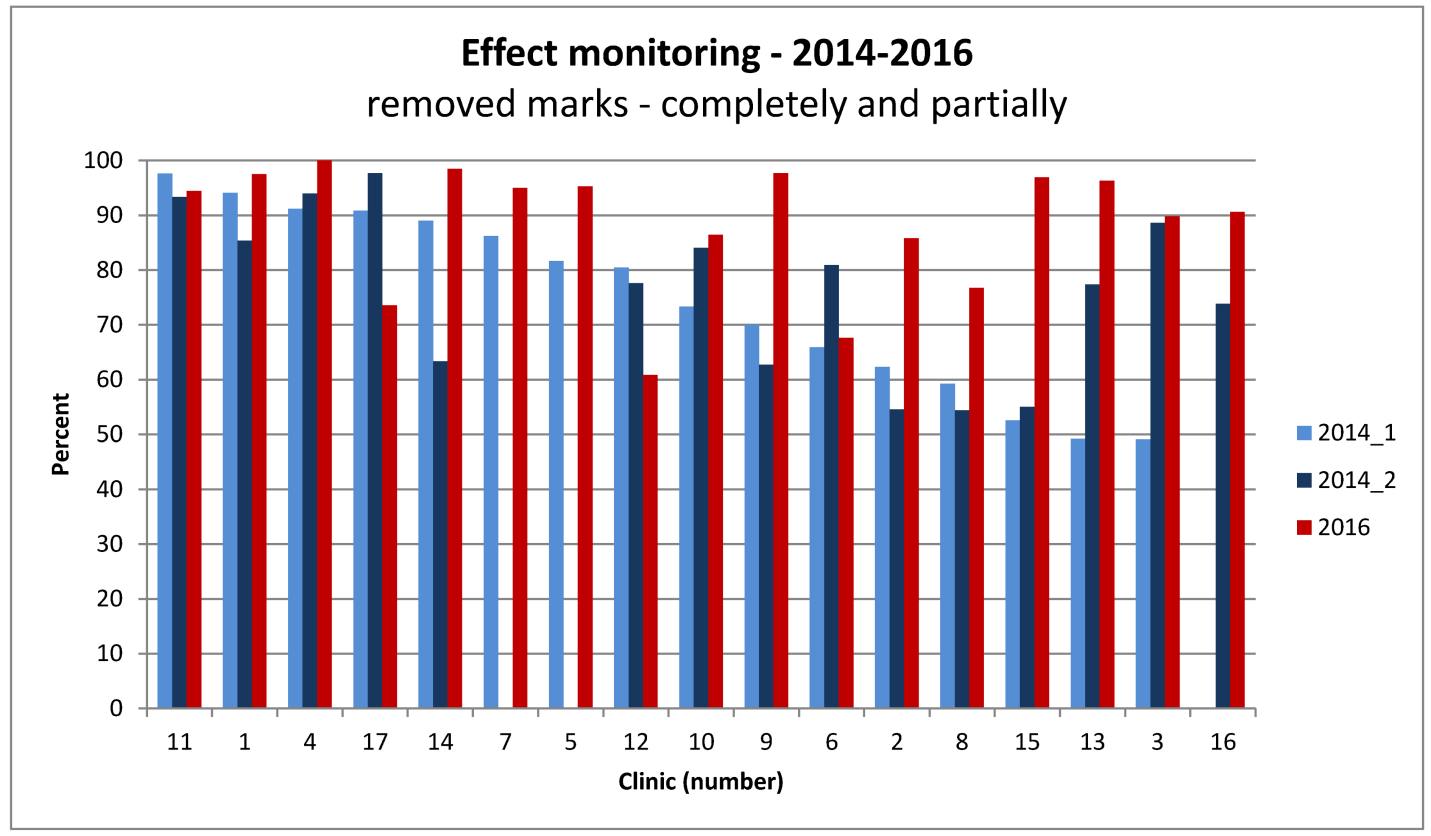
Monitoring the outcome quality of cleaning and disinfection in hospitals in Frankfurt/Main, 2014 and 2016, by the glow-check method according to the different sites – percent compliance in different hospitals

For monitoring the effect of cleaning and disinfection, the glow-check method was used. When scoring complete removal of the mark as two points and partial removal by one point, in total, 87.8% of the maximum number of points possible were reached in 2016. This is a good improvement compared to 2014, when a compliance of 75% was observed. In 2016, compliance in the different hospitals ranged from 67–100%, compared to 49–97% in the initial examination in 2014. Especially in the hospitals with bad compliance in 2014, improvements were demanded and achieved (Figure 3 (Fig. 3)).

**Figure 4:**
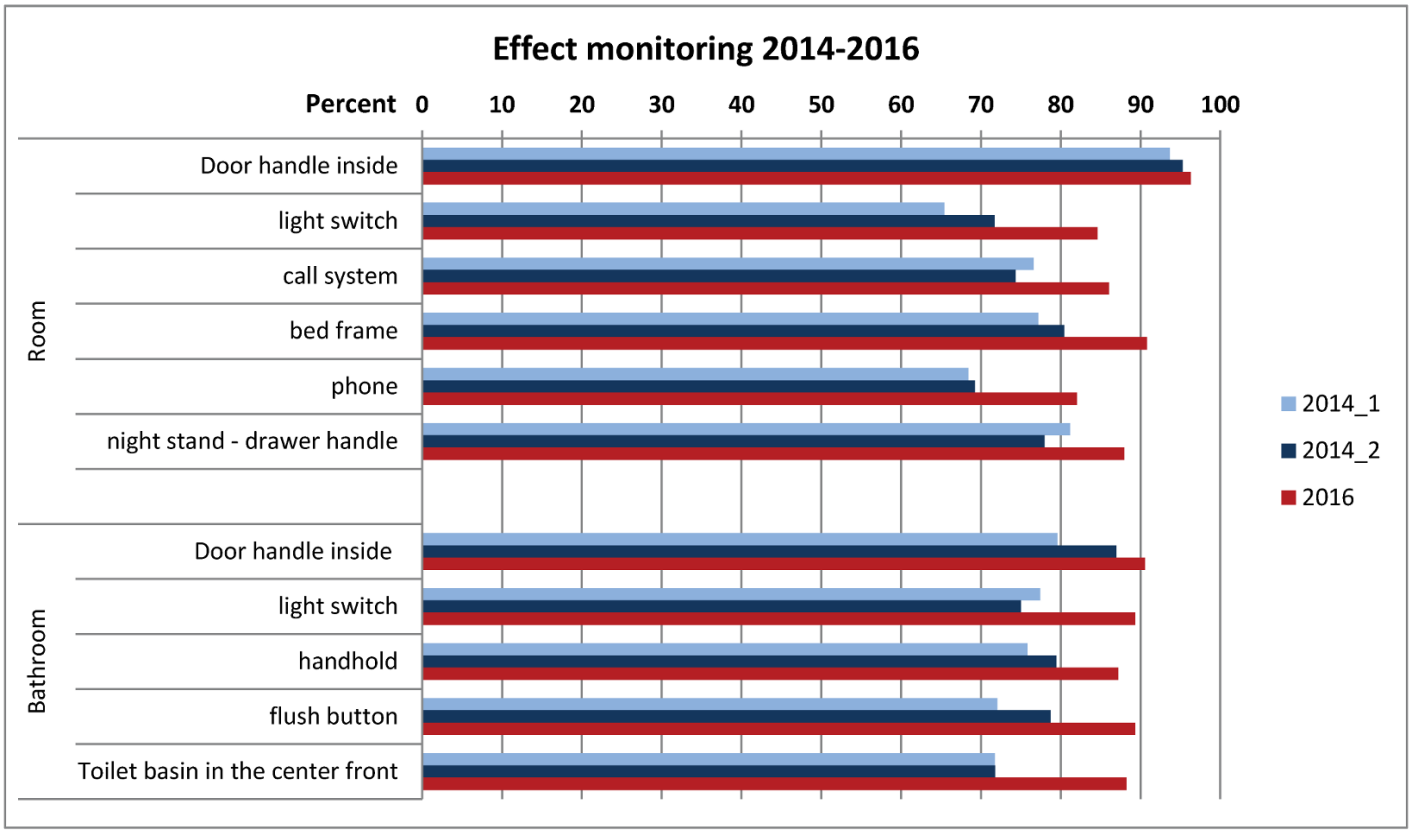
Monitoring the effect of the cleaning and disinfection in hospitals in Frankfurt/Main, 2014 and 2016, by the glow-check method on different sites – percent of properly cleaned sites

Regarding the various marked sites, the best improvements were seen at those points which had been poorly reprocessed in 2014 (Figure 4 (Fig. 4)). Hence, the results of the observation for process quality are confirmed. 

In Table 3 (Tab. 3), the results of this survey are compared with data from the literature [9], [11], [12], [13], [14], [15], [16], [17]. For comparison reasons, only the full removal of the marks was taken into account. In 2016, 85% of the marks in the Frankfurt hospitals was removed completely, resulting in a 22% improvement compared to the first survey in 2014 [9]. In the other studies published, a maximum of 85% compliance was also found. It is striking that in the other studies, however, improvement rates of up to 57% were found [11], [12], [13], [14], [15], [16], [17]. In Frankfurt, a 22% increase was observed due to better compliance in the initial examination in 2014 (63% vs 23–47% in other studies).

## Discussion

Postal surveys are often carried out on hygiene in hospitals. These can usually record the structural quality only. Good structural quality (i.e., personnel, equipment, training, and control of employees) is a necessary but not sufficient prerequisite for good hygiene. Good structural quality does not automatically guarantee good process quality. In the event of staff shortage, illness, frequent personnel changes, and lack of training and control of the employees, the process quality can be insufficient despite good structural quality. In an earlier study by the Health Office on surface reprocessing in nursing homes, only small and not significant correlations between structural quality on the one hand and process and outcome quality on the other were found [18]. Therefore, for assessing the quality of hygiene, it is also important to check the process and outcome quality.

This was done in the present survey. Before discussing the results, however, the limitations should be addressed. Regarding process quality, detailed observations of the processes were carried out. Whereas the process quality of automatic procedures can be monitored easily by monitoring the technical data, all manual procedures, such as hand hygiene and surface preparation, including manual steps in medical device preparation, must be observed. Thus, an observer effect cannot to be excluded; on the contrary, such an effect is very probable. Hence, the process quality of surface reprocessing can only be assessed with restrictions. A Hawthorne effect has to be taken into account. The process quality may also be influenced if – in the case of announced examinations of the public health department – only selected personnel is provided, or especially carefully prepared.

The examination of the quality of outcome, defined as the prevention of infections, is virtually impossible, since prevented infections cannot be shown statistically, or at best can only be statistically represented by large numbers of cases. Attributing it to individual factors such as hand hygiene, surface cleaning and disinfection or reprocessing of medical devices is even less feasible. The outcome quality defined as the outcome of processes, e.g., sterilization of medical devices or preparation of surfaces, can be measured. For the verification of the surface preparation markings with fluorescent dyes, the ATP test and cultural impact methods are available [10]. All procedures have their advantages and limitations. Microbiological detection by means of microbiological samples is considered to be the gold standard, but the procedure is expensive and the results are available only after several days. The ATP method can detect bacterial adenosine triphosphate; it is fast but relatively expensive. The fluorescence method, i.e., using a pen, stamp or spray to mark a surface with a fluorescent dye, and checking with a black light lamp whether this mark has been removed by the cleaning process, is very simple, comparatively inexpensive and is suitable for directly presenting the cleaning performance. – That is, it is suitable for direct feedback and training purposes. The disadvantage is that the method only shows the quality of the cleaning (wiping with pressure) and gives no indication of the quality of disinfection. In control situations as in the present study, the result can theoretically be falsified by the fact that the marked sites were previously known to the employees of the cleaning services or the cleaning personnel themselves checked the markings with a small black light lamp of their own and then cleaned them more intensively.

In summary, the data on structural quality, process quality and outcome quality can be influenced by the institutions surveyed. Process observations are generally subject to observer bias, and all the indicators for quality assessment, including the fluorescence method used in this study, have their methodological limits. In addition, the inspections carried out by the health office can in principle only reflect the actual moments of inspection. Against this background, the legislator in Germany has rightly pointed out the responsibility of the institutions and the individuals themselves (§1 [19]) . 

In general, the effect of a single measure, e.g., the disinfection of critical surfaces in the patient’s room or the reprocessing of the bathroom cannot be demonstrated directly by rates of pathogen transmission or nosocomial infections. This can only be achieved within the scope of a controlled study, if all other hygienic processes (hand hygiene, medical product reprocessing, operating procedures, etc.) are controlled. Direct comparison with wound infections or device-associated infections, which have to be monitored in the hospitals [19] [§23], or with multi-drug resistant pathogens, which must be reported to the public health service [20], [21], is not suitable for this purpose because of many other additional influencing factors. 

Therefore, the aim of our inspections could not be to achieve a demonstrable effect on the transmission and infection rates in the hospitals. The aim was to monitor and improve the hygienic process and to raise awareness of the importance of surface preparation. 

This has been achieved. The clinics improved the quality of the structure (training of the foremen, interface plan, increase of the stipulated cleaning performance on weekends and holidays). Nevertheless, we see further need for improvement. The contractually provided time for the reprocessing of the bathrooms must be increased in general, since in both surveys, in 2014 and in 2016, the proper cleaning and disinfection of the bathrooms could not be completed in any clinic within the time commissioned. Additionally, from a hygienic view, complete cleaning restricted to certain days appears incomprehensible and unacceptable. 

Significant improvements between 2014 and 2016 were also achieved in terms of process quality. The critical points were cleaned better, and the improvement was particularly good in those points which had previously been badly reprocessed. In accordance with the observed process quality, the clinics also improved the quality of the outcome. More marked points were cleaned appropriately, and again, the greatest improvements were obtained in the previously poorly prepared areas.

In various other investigations, the quality of the cleaning results measured by the fluorescence method were significantly improved by targeted interventions. In others studies, a decrease in the germ load, for example, with methicillin-resistant *Staphylococcus aureus* strains [13], and a reduction of colonizations or infections with multi-drug resistant pathogens [22], [23] were demonstrated. In a further study, a significant reduction in infections were achieved by additional cleaning staff, which resulted in an estimated savings of 30,000–70,000£ to the clinic despite increased personnel expenses [23].

Inadequate cleaning can not only increase the risk of nosocomial infections and colonization with multi-drug resistant pathogens. Further negative consequences are, for example, damage to the image of the hospital, when patients complain about dirt and insufficient hygiene. According to DGKH cleaning is one of the very few areas that the patient can observe and also assess so that a hospital could profit from a qualified cleaning [24]. Other possible consequences are legal actions because of infectious disease caused by insufficient cleaning and material damage to furniture and buildings [5]. The DGKH notes, however, that the great importance of cleaning does not correspond to the fact that the cleaning service only has low social standing and that in the frequently outsourced cleaning companies, untrained employees, low salaries, and frequent job changes dominate.

Against this background, rethinking was demanded of the hospital management, as was qualification of the specialist for hospital cleaning, for instance, and a higher appreciation and estimation of the cleaning service [25]. Corresponding curricula are already established in France [2]. Within the framework of a bundle strategy, however, not only knowledge and skills must be communicated to the cleaning staff, but feedback must be given on the quality of their work. Cleaning staff must know and feel that they are part of a team in the hospital and that they have an important and by no means negligible part in the care of patients when they clean and disinfect the surfaces [25], [9], [17].

New approaches to reduce germ load on surfaces, such as “self-disinfecting” surfaces by using surface materials which are either coated or mixed with metals (e.g., silver or copper) or with microcidal substances (e.g., triclosan or quarternary ammonium phosphates) are currently under investigation as a further option for the improvement of hygiene in the patient environment. Whether they meet the expectations remains to be proven, since there is still insufficient practice experience [26], [27].

All hygiene measures are only as good as the weakest link in the chain. The importance of surface preparation – with regard to the often very long persistance of germs on inanimate surfaces [28] and especially with regard to the increase of multidirectional pathogens – should be given more attention [1], [2].

## Notes

### Competing interests

The authors declare that they have no competing interests.

## Figures and Tables

**Table 1:**
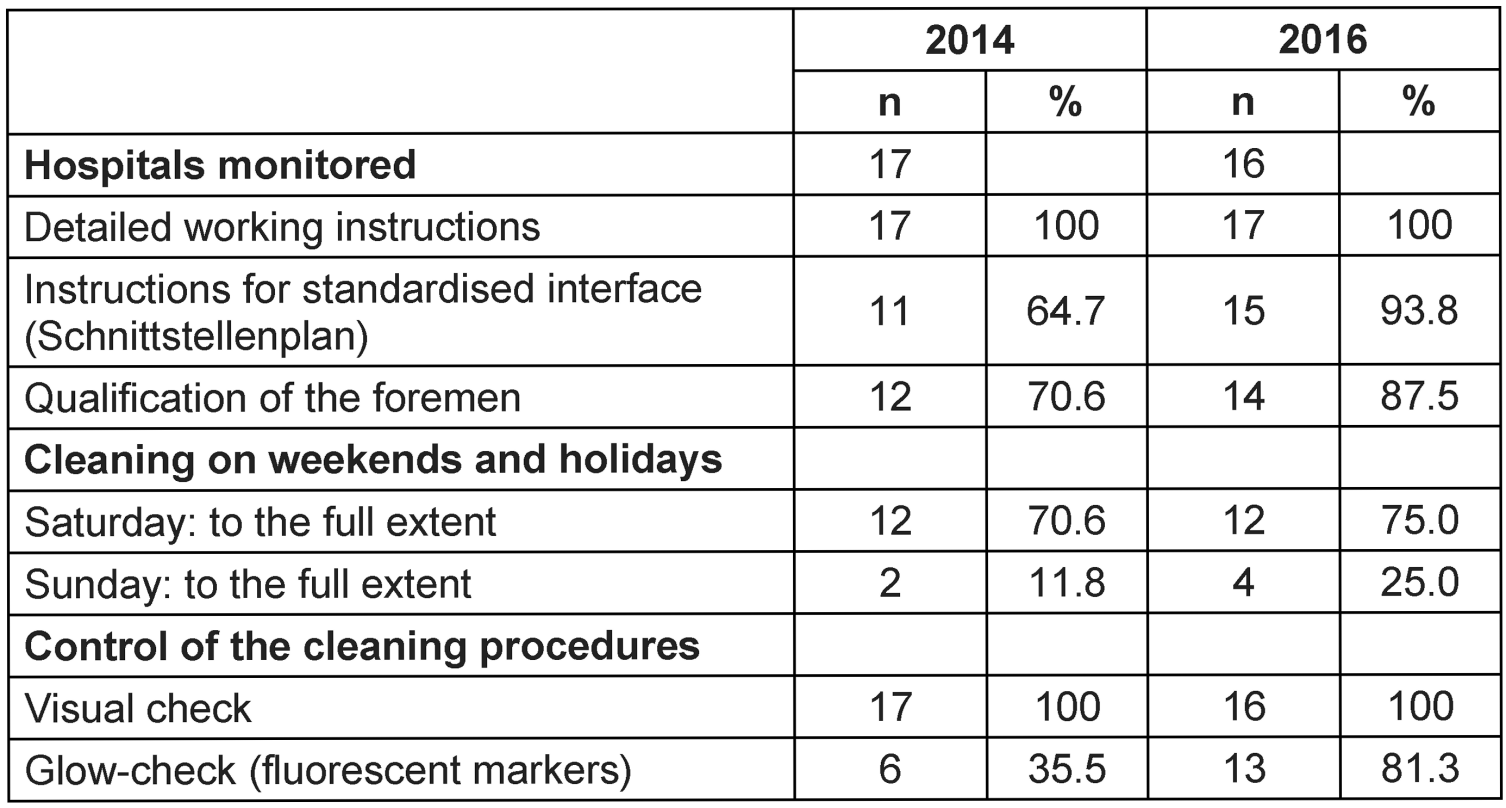
Structural quality for the cleaning and disinfection of surfaces in hospitals in Frankfurt/Main, 2016 vs 2014

**Table 2:**
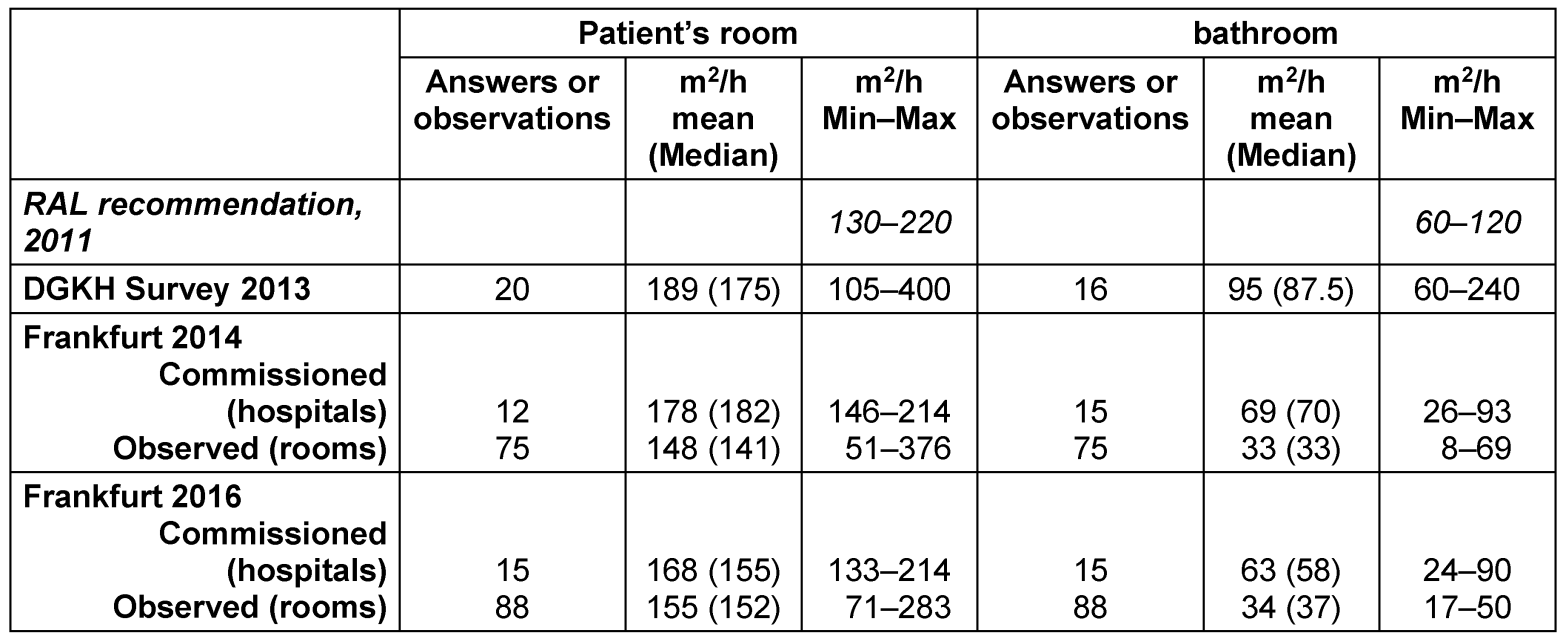
Time for cleaning and disinfection of patient rooms and bathrooms – recommendation of the RAL, questionnaire survey of the German Society for Hospital Hygiene (DGKH) 2013, and the data of Frankfurt hospitals 2014 and 2016

**Table 3:**
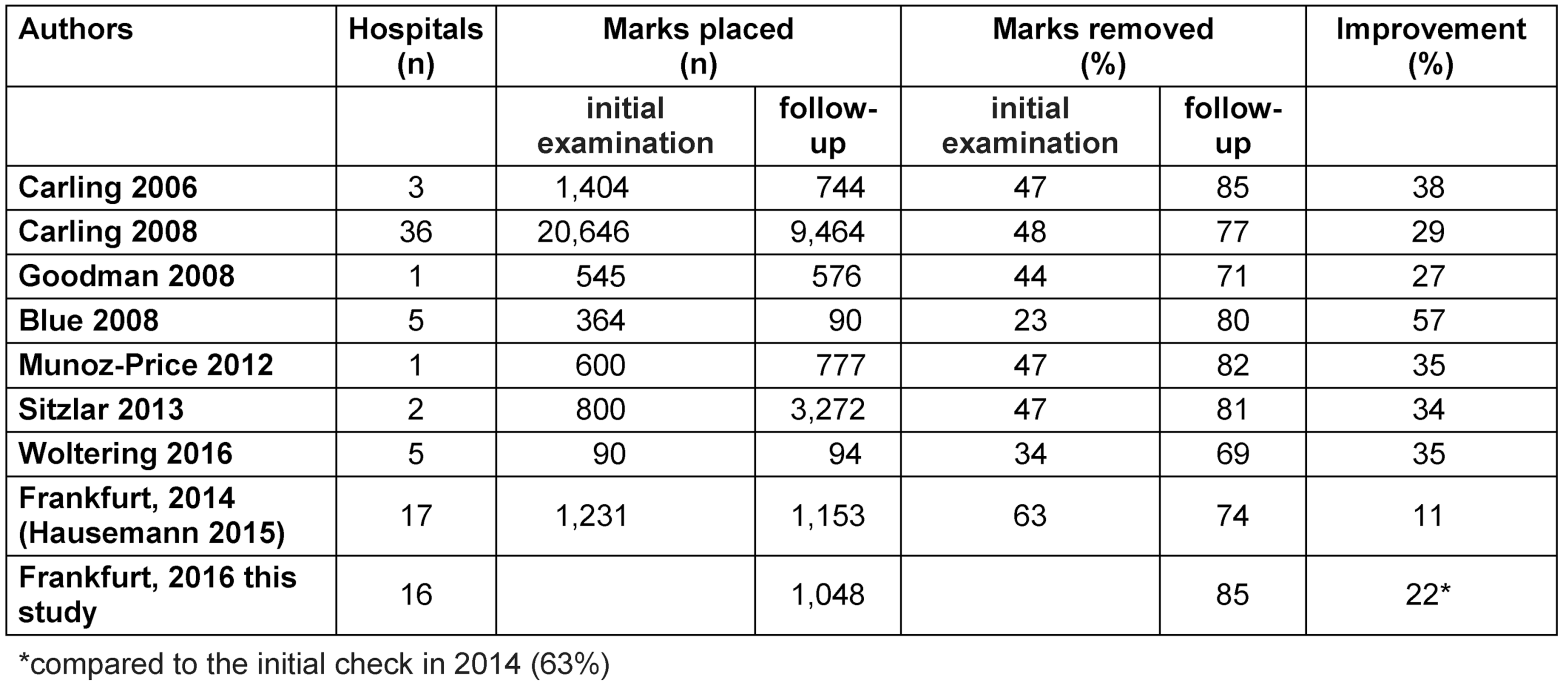
Effect of hospital cleaning as assessed by the glow-check method. Results from the hospitals in Frankfurt 2014 and 2016 compared to published data from other studies (only completely removed marks taken into account)
